# Individual and community level determinants of childhood full immunization in Ethiopia: a multilevel analysis

**DOI:** 10.1186/s12889-015-2315-z

**Published:** 2015-09-28

**Authors:** Samir A. Abadura, Wondwosen T. Lerebo, Usha Kulkarni, Zeleke A. Mekonnen

**Affiliations:** Oromia Regional Health Bureau, Addis Ababa, Ethiopia; Department of Public Health, Mekelle University College of Health Sciences, Mekelle, Ethiopia; Health Promotion and Disease Prevention Directorate, Federal Ministry of Health, Addis Ababa, Ethiopia

**Keywords:** Multilevel, Community level, Individual level, Ethiopia, Ethiopian Demographic and Health Survey, Full childhood immunization, Expanded program on immunization

## Abstract

**Background:**

Expanded program on immunization is one of the most successful and cost effective public health interventions that protect children against vaccine preventable diseases. The full childhood immunization coverage in many parts of Ethiopia is far from optimal. Hence, the main objective of this study was to assess factors associated with childhood full immunization in Ethiopia.

**Methods:**

The data source for this study was the 2011 Ethiopian Demographic and Health Survey. Multilevel regression analysis techniques were used to conduct the analysis. Accordingly a two level multilevel regression analysis model was built with individuals (level 1) nested with in communities (level 2).

**Results:**

A total of 4983 children aged 12–59 months nested within 520 clusters were included in the analysis. According to the analysis results, in the year 2011, 26 % of children less than 5 years old were fully immunized in Ethiopia. Being born at health institutions, higher level of maternal education, media exposure, region of residence and residing in communities possessing higher maternal antenatal care services utilization were positively associated with childhood full immunization. In contrary to this, the number children aged less than 5 years in the household was negatively associated with childhood full immunization. The random effect results indicated that 21 % of the variation among the communities was due to community level factors.

**Conclusions:**

It was found that various individual and contextual factors were associated with childhood full immunization. In addition, significant community level variation remains after having controlled individual and community level factors which is an indicative of a need for further research on community level factors. Hence, utilizing multilevel modeling in determining the effect of both individual and contextual level factors simultaneously had brought an important output which may help planners, policy and decision makers to emphasize on both individuals and communities in which they live.

## Background

The Expanded program on immunization (EPI) was established in 1974 to build on the success of the global smallpox eradication program and to ensure that all children in all countries benefited from life saving vaccines [1]. It is a global effort of Governments, United Nations agencies and Non-governmental Organizations (NGOs) to immunize the world’s children to prevent the suffering, disability and death due to Vaccine preventable diseases (VPDs), which include Measles, Diphtheria, Whooping Cough, Tetanus, Tuberculosis, Polio, Chickenpox, Haemophilus Influenza Type b, Hepatitis A, Hepatitis B, Influenza, Mumps, Pertusis, Pneumococcus, Rotavirus, and Rubella [2, 3]. Globally immunization coverage continues to increase dramatically [4]. Despite the overall success of immunization programs to date, it is estimated that almost 11 million children under 5 years of age continue to die each year [3]. Especially in developing countries, it is still far from the universal target, leading to preventable mortality. According to the World health organization (WHO), childhood vaccinations could have prevented an estimated 2.9 million deaths in children in 2007 [4].

In Ethiopia EPI program was initiated by the Ministry of health (MoH) in the year 1980, with the target of reaching 90 % coverage among children less than 1 year of age by the year 1990 [5]. From there on wards routine immunization services have been provided to children less than 1 year of age for the six major VPDs (tuberculosis, poliomyelitis, tetanus, diphtheria, pertusis and measles) [6, 7]. Starting from 2011 vaccine against Pneumococcus was under implementation in addition to the major ones which will some up to 9 VPDs [8].

Ethiopia is an ancient country situated in the horn of Africa, having great geographical diversity with a projected population size of 79.8 million in the year 2010. Women in the reproductive age constitutes 24 % of the population; about 5/6 of the population live in rural areas. According to Ethiopian Demographic and Health Survey (EDHS) 2011 report vaccination coverage rates for the various childhood vaccines in Ethiopia are the lowest amongst developing countries and far from countries target (90 %) in the year 2010 [9].

The driving forces towards the utilization of immunization services includes, a clear understanding of the benefits of vaccination among community members, a readiness for providing vaccination by the health services, and interventions to overcome barriers to immunization services [7, 10]. Increasing immunization coverage for childhood diseases has become an important developmental issue [11]. It is estimated that about 27 million children and 40 million pregnant women do not receive the full complement of vaccines, out of which over 2 million people die worldwide yearly from VPDs [12]. Vaccine Preventable illnesses constitute major causes of morbidity and mortality in Africa [13].

Previous studies had identified factors associated with childhood full immunization which include, child place of delivery [14–18], age of child [17, 19, 20], birth order of the child [15, 18, 21, 23–25], number of under 5 years children in the household [22], maternal age [14, 19, 20], parental education [7, 18–20, 22, 23, 25–27], family wealth index [13–15, 23, 25, 28], parental employment status [19, 29], maternal marital status [15, 17, 19, 25], antenatal care (ANC) and tetanus toxoid (TT) [17], religious affiliation [14, 26, 28], and exposure to mass media [13] and at the community level place of residence [7, 17, 19, 20, 23, 26, 29, 30], geographic region [21, 23, 26, 31–33], community maternal hospital delivery [21, 31], community/neighborhood poverty [33], community maternal unemployment and illiteracy [19], distance from health facility [31] and community maternal ANC services utilization were found to be associated with immunization status.

Although several studies had examined and documented the determinant factors associated with childhood immunization in Ethiopia, the influence of contextual factors on the immunization status of children had received less consideration. In addition to these, using a single level logistic regression analysis technique to analyze a data that has a hierarchical structure (i.e. children nested within communities) violates the independence assumptions of regression [34, 35]. Hence to address these limitations, and to further document the significant effect of individual and contextual level factors in the field of public health, this study takes a step further, utilizing a multilevel logistic regression modeling techniques. Therefore, the purpose of this study was to determine both individual and community level factors that are associated with childhood full immunization in Ethiopia. The findings from this study will help planners, policy and decision makers to have good insight of determinants of childhood immunization and take appropriate measures to strengthen immunization services.

## Methods

### Data source

The dataset used for this analysis was the 2011 EDHS. It was conducted by the CSA under the auspices of FMoH with technical assistance from ICF International through its MEASURE DHS project. The survey was representative of both the national and regional levels. It is the latest and third nationally large scale dataset on demographic and health information collected using cross sectional survey methods. This study analyzed the sample of mothers aged 15–49 years who had at least one child aged 12–59 months in the last 5 years (2005–2010).

### Study variables

#### Outcome variable

The outcome variable in this study was the probability of the last child 12–59 months of age being fully immunized. This was assessed by the interviewer based on the report on the immunization card and from mothers’ verbal reports. It was categorized as Yes if the child was fully immunized (A dose of BCG, 3 doses of DPT, 3 doses of Polio and a dose of Measles) and No otherwise.

#### Independent variables

Consistent with the research objective and given the effect of the outcome variable at more than one level where children were nested within communities, two levels of explanatory variables were considered. Level 1 contained individual characteristics (of both child and parents) and that of the community at level 2, which included the characteristics of the communities in which each child resided. Community level variables used in the analysis were from two sources; direct community level independent variables which were used without manipulation and aggregate community level variables which were aggregates of individual level independent variables.

### Data analysis

#### Descriptive statistics

For the whole analysis STATA 12 software package was used. Prior to the commencement of the analysis data cleaning, labeling, coding and recoding were done for all selected variables. Categorization was done for continuous variables using information from different literatures and re categorization was done for categorical variables accordingly. Frequency and percentage was used to report categorical variables, while median followed by Inter quartile Range (IQR) was used to report non parametric continuous explanatory variables. In addition cross tabulation was used to show the proportion of different categories of each characteristic with respect to childhood full immunization.

Initially, in the child data set there were 5842 children distributed among 595 clusters. For the purpose of analysis age of the child (12–59 months) and number of children per cluster at level 2 was used as criteria to create the full data set. Based on these criteria 896 children were excluded from the analysis. Hence, the final data set used in the analysis comprises 4983 children distributed in 520 clusters [34, 35].

#### Bivariate multilevel regression analysis (MLRA)

The effect of each individual and community level predictor variables on the outcome variable were checked at significance level of 0.05 and 95 % CI, independently. Variables which were statistically significant at the bivariate multilevel logistic regression analysis were considered as candidates for the individual and community level model adjustments.

#### Model specification (multivariate MLRA)

This study applied multilevel analysis techniques in order to account for the hierarchical nature of the DHS data and the binary response of the outcome variable. A two level MLRA was applied in this study in which individuals (level 1) were nested with in communities (level 2). The level 1 model represents the relationships among the individual level variables and the outcome variable while the level 2 model examines the influence of community level factors on the outcome variable. For the bivariate and multivariate multilevel logistic regression analysis the STATA syntax *xtmelogit* was used [35].

The Final two level model is:

*logit* (*πij*) = *log* [*πij*/(*1* − *πij*)] = *β0* + *β1xij* + *β2xij* …. +*u0j* + *e0ij* [34, 35].

Where, πij: probability of *i*th child in the *j*th community to be fully immunized, β0: intercept, βn:regression coefficient, Xij: independent variables, u0j: community level errors and e0ij:individual level errors.

Four models containing variables of interest were fitted. *Model 1*: It is the null model, used to check the variability among the communities without inserting a study variable. It contained no exposure variable. It’s the first step in MLRA used to provide evidence whether the data has a justifiable evidence to assess the random effects at the community level. *Model 2*: It is a multivariate model adjustment for individual level variables which were significant at the bivariate MLRA level. It was built by stepwise regression analysis technique which enables removing independent variables that become insignificant during insertion of every other independent variable to the model. Hence, independent variables which were significant in model-2 (i.e. Individual level variables model adjustment) were considered as candidates of the final model. *Model 3*: It is a multivariate model adjustment for community level variables which were significant at the bivariate MLRA level. Similarly, Model 3 was built by stepwise regression analysis technique. All independent variables which were statistically significant in model-3 (community level variables model adjustment) were included in the final model as potential candidates. *Model 4*: It is a multivariate MLRA model adjustment of the outcome variable against predictor variables which were statistically significant either at individual or community level model adjustments.

#### Parameter estimations

The measures of association (fixed-effects) estimates the associations between likelihood of children to be fully vaccinated and various explanatory variables expressed as Adjusted Odds Ratio (AOR) with their 95 % Confidence Intervals (CIs). The measures of variation (random-effects) were reported as Intra community correlation coefficient (ICC) which is the percentage variance explained by the higher level (community level variables) [36]. Proportional Change in Community Variance (PCV), expresses the change in the community level variance between the empty model (Model 1) and the consecutive models [36].

#### Multicollinearity and interaction

The presence of multicollinearity was checked among independent variables using Variance Inflation Factor (VIF) at cut off point of 10. Predictors having a VIF value of less than 10 indicate absence of multicollinearity. Similarly, interactions between individual and community level characteristics were added to the models to test whether the community level characteristic effects on full immunization were modified by individual level characteristics [37].

#### Model fit statistics

Akakie Information Criterion (AIC) was used to estimate the goodness of fit of the adjusted final model in comparison to the preceding models (individual and community level model adjustments). The AIC value for each subsequent model was compared and the model with the lowest value was considered to be the best fit model [35, 38, 39].

#### Ethical approval

Permission to use the EDHS data in this study was obtained from ORC Macro Inc, Chicago and Mekelle University College of Health Sciences Department of Public Health. The ORC Macro Inc has removed all information that could be used to identify the respondents. Hence, no effort was made to identify any household or individual respondent interviewed in the survey.

## Results

### Univariate analysis results

The individual and contextual level background characteristics of the study participants were showed in Tables 1 and 2. Overall, a total of 4983 children at level 1 nested within 520 clusters at level 2 were included in the analysis. Table 1 showed that, nearly 26 % of the children aged 12–59 months had been fully immunized at the time of survey. Almost half of the households (50 %) were with poorest and poor wealth index. Only 15 % of mothers responded that they decided by themselves on their health matters. The median number of under 5 years children found in each household was found to be 2 (IQR = 1).

**Table 1:** Frequency and percentage distribution of individual level background characteristics of mothers and their children aged 12–59 months, EDHS 2011 (*n* = 4983)

Characteristics		Number	Percent (%)
Fully immunized	No	3701	74.3
	Yes	1282	25.7
Children age group (months)	12–23	955	19.2
	24–35	1207	24.2
	36–47	1431	28.7
	48–59	1390	27.9
Sex of child	Female	2436	48.9
	Male	2547	51.1
Children birth order	1st	909	18.3
	2nd–4th	2224	44.6
	≥5th	1850	37.1
Child place of birth	Home	4498	90.3
	Health institution	483	9.7
	Missing	2	0.0
Mothers age group (years)	≤24	1048	21.0
	25–34	2650	53.2
	≥35	1285	25.8
Fathers age group (years)	≤24	151	3.0
	25–34	1701	34.0
	≥35	2720	55.0
	Missing	411	8.0
Maternal educational level	No education	3561	71.5
	Primary	1253	25.1
	Secondary and above	169	3.4
Fathers educational level	No education	2657	53.3
	Primary	1846	37.0
	Secondary and above	459	9.2
	Missing	21	0.4
Sex of head of household	Female	897	18.0
	Male	4086	82.0
Marital status	Never in union (single)	21	0.4
	Living together	4588	92.1
	Not living together	374	7.5
Maternal ANC utilization	No	1673	33.6
	Yes	1242	24.9
	Missing	2068	41.5
Maternal TT utilization	No	1448	29.1
	Yes	1467	29.4
	Missing	2068	41.5
Religious affiliation	Orthodox	1535	30.8
	Muslim	2242	45.0
	Protestant	1050	21.1
	Traditional and others	156	2.1
Mothers occupation	Did not work	2521	50.6
	Non professional	1521	30.5
	Professional	887	17.8
	Missing	54	1.1
Fathers occupation	Did not work	110	2.2
	Non professional	4104	81.6
	Professional	769	16.2
Wealth index	Poorest	1472	29.5
	Poor	1027	20.6
	Medium	886	17.8
	Rich	902	18.1
	Richest	696	14.0
Media exposure	Not exposed	4033	80.9
	Exposed	950	19.1
Maternal health care decision making	Husband/partner alone	1283	28.0
	By her self	689	15.0
	Jointly with her husband	2610	57.0
Number of under five child		Median	2 (IQR = 1)

**Table 2:** Frequency and percentage distribution of contextual level background characteristics of mothers and their children aged 12–59 months, EDHS 2011 (*n* = 4983)

Characteristics		Number	Percent
Place of residence	Rural	4372	87.7
	Urban	611	12.3
Region of residence	Tigray	474	9.5
	Affar	337	6.7
	Amahara	697	14.0
	Oromia	792	15.9
	Somali	337	6.7
	BEGU	499	10.0
	SNNP	845	17.0
	Gambella	388	7.8
	Harari	286	5.8
	Addis Ababa	45	0.9
	Dire Dawa	283	5.7
Distance from health facility	Big problem	3778	75.8
	Not big problem	1202	24.1
	Missing	3	0.1
Transportation to health facility	Big problem	3944	79.1
	Not big problem	1036	20.8
	Missing	3	0.1
Community media exposure	Low	4536	91.0
	High	447	9.0
Community poverty	Low	2506	50.3
	High	2477	49.7
Community maternal ANC utilization	Low	2527	50.7
	High	2456	49.3
Community institutional delivery	Low	4678	93.9
	High	305	6.1
Community maternal unemployment rate	Low	2676	53.7
	High	2308	46.3
Community maternal illiteracy rate	Low	1048	21.0
	High	3935	79.0

As depicted in Table 2, nearly 88 % of the participants were rural residents. At the time of the survey, 94 % of mothers were dwelling in communities with low community institutional delivery. Majority (79 %) of mothers were residing in communities having high proportion of community illiteracy.

### Multivariate MLRA results

Table 3 depicted multilevel multivariate logistic regression analysis results for odds of childhood full immunization. The Model 1 (null model) showed that there was a significant variability in the odds of childhood full immunization across communities [τ = 1.42, *p* < 0.001]. As indicated in the ICC, 30 % of variability in the odds of a child being fully vaccinated is due to community level factors.

**Table 3:** Multivariate MLRA of individual and community level factors with childhood full immunization among 12–59 months Ethiopian children, EDHS 2011

Individual and community level characteristics		Model 1	Model 2	Model 3	Model 4
			AOR [95 % CI]	AOR [95 % CI]	AOR [95 % CI]
Fixed effects					
Child place of birth	Home	–	1	–	1
	Health institution	–	1.54 [1.173–2.047]	–	1.40 [1.058–1.859]
Maternal education	No education	–	1	–	1
	Primary	–	1.43 [1.201–1.725]	–	1.42 [1.191–1.710]
	Secondary +	–	1.88 [1.210–2.922]	–	1.93 [1.242–3.000]
Religious affiliation	Orthodox	–	1	–	–
	Muslim	–	0.70 [0.554–0.906]	–	–
	Protestant	–	0.62 [0.470–0.843]	–	–
	Traditional/others	–	0.48 [0.277–0.862]	–	–
Household wealth index	Poorest	–	1	–	1
	Poor	–	1.29 [1.030–1.634]	–	1.30 [1.034–1.643]
	Medium	–	1.23 [0.967–1.587]	–	1.23 [0.963–1.585]
	Rich	–	1.31 [1.016–1.689]	–	1.32 [1.023–1.706]
	Richest	–	1.83 [1.337–2.521]	–	1.62 [1.172–2.245]
Number of U5 children		–	0.86 [0.775–0.948]	–	0.87 [0.789–0.964]
Media exposure	Not exposed	–	1	–	1
	Exposed	–	1.23 [1.009–1.522]	–	1.25 [1.019–1.539]
Residence place	Rural	–		1	–
	Urban	–		1.50 [1.072–2.118]	–
Geographic region	Affar	–	–	1	1
	Tigray	–	–	6.50 [3.649–11.544]	6.36 [3.550–11.934]
	Amahara	–	–	2.51 [1.446–4.383]	2.55 [1.459–4.478]
	Oromia	–	–	1.15 [0.655–2.033]	1.14 [0.664–2.019]
	Somalia	–	–	1.86 [0.974–3.577]	2.37 [1.232–4.575]
	BEGU	–	–	2.34 [1.304–4.228]	2.42 [1.337–4.385]
	SNNP	–	–	1.54 [0.884–2.684]	1.49 [0.852–2.611]
	Gambella	–	–	1.65 [0.900–3.032]	1.51 [0.815–2.824]
	Harari	–	–	1.40 [0.719–2.757]	1.46 [0.751–2.874]
	Addis Ababa	–	–	7.34 [2.823–19.105]	5.39 [2.047–14.195]
	Dire Dawa	–	–	3.71 [1.941–7.094]	3.89 [2.014–7.515]
Community poverty	High	–	–	1	–
	Low	–	–	1.70 [1.313–2.203]	–
Community ANC utilization	Low	–	–	1	1
	High	–	–	1.54 [1.205–1.990]	1.50 [1.177–1.934]
Random effects					
Community variance (SE)		1.42* (0.17)	1.12* (0.14)	0.82* (0.11)	0.85* (0.12)
ICC (%)		30.14	25.39	19.95	20.53
PCV (%)		Ref	21.12	42.25	40.14
Model fit statistics					
Log-likelihood		−2647.38	−2578.76	−2559.80	−2532.20
AIC		5298.77	5185.54	5149.61	5108.40

*P-value < 0.05

The variation in full immunization in Model 2 remained significant [τ = 1.12, *p* < 0.001], with 25 % of variance among observations being attributed to community level factors. Table 3 (Model 2) also revealed that the PCV for individual level factors model adjustment was 21 %; indicating that 21 % of the variance in the odds of childhood full immunization between communities was explained by individual level factors found in the model (model 2).

Random parts of Model 3 depicted that the unobserved community level variability in odds a child to be fully immunized remained significant [τ = 0.82, *p* < 0.001] with 19.95 % of the variance among the communities was due to community level factors. Similarly, the PCV indicated that almost 42 % of the variance in the likelihood of full immunization across communities was explained by the community level factors found in the model (model 3).

After adjusting for other variables at both individual and community levels, those children delivered in the health institutions had 40 % [AOR = 1.40, 95 % CI 1.058–1.859] higher likelihood of being fully immunized as compared to those who were delivered at home. Mothers with secondary and above educations had 93 % [AOR = 1.93, CI 1.242–3.000] higher odds of fully immunizing their children than the uneducated mothers. As wealth index of households increase and the mother has media exposure, the odds of completing childhood vaccination increases. In contrary to this when the number of children in the household increased by one child, the chance decreases significantly (Table 3).

After controlling for other individual and community level variables, children from communities with high proportion of community maternal ANC services utilization rates had 50 % [AOR = 1.50, 95 % CI 1.177–1.934] higher chance of being fully immunized than those from communities with low community maternal ANC services utilization.

Reasonably, the variance at the community level in Model 4 (Table 3) remained significant [τ =0.85, *p* < 0.001] even after controlling for both individual and community level factors. The ICC value was 20.53 %, showing that only 20.53 % of the variation among the clusters was due to community level factors. The ICC at model 4 decreased to 20.53 % indicating that the inclusion of community level variables was important for obtaining a better explanatory model. As shown in the PCV, almost 40 % of variance in the odds of full immunization across communities was due to simultaneous effects of both individual and community level factors found in model 4 (final model).

### Multicollinearity and interaction

The mean VIF value was 1.62 indicating the absence of multicollinearity among the predictor variables found in the model. The presence of interaction among explanatory variables was checked and there was no significant interaction between the individual and community level variables.

### Model fit statistics

As shown in Table 3 (model fit statistics), the values of AIC showed subsequent decrease which indicated that each model represents a significant improvement over the previous model and it points out the goodness of fit of the final model built in the analysis.

## Discussion

For this particular study 4983 children nested within 520 clusters were included in the analysis. The results of the study showed that individual and community level variables were the major predictors of childhood full immunization status among children of Ethiopia based on the data from 2011 EDHS.

Based on the results of analysis, by the year 2011 the childhood full immunization coverage of Ethiopia was almost 26 %. This result was a little bit higher than the EDHS 2011 report which was 24 % national childhood full immunization coverage. The possible explanation for this discordance might be the difference in sample size.

Evidence of a strong statistical association was found between childhood full immunization status and place of delivery. According to the results of this study, children born at health institutions were found to be more likely to be fully immunized than those delivered at home. This finding is in line with previous studies conducted elsewhere [15–19, 40]. This may be due to the fact that mothers who gave birth at health institution were closer to health services and most of the time first dose of vaccination (OPV 0) is given just after birth and parents will be educated regarding subsequent vaccinations [41].

In this study maternal education was an important predictor variable of childhood full immunization status; showing that educated mothers had significant chance of fully immunizing their children than the uneducated mothers. This finding is consistent with the findings of other multilevel analysis studies conducted in 24 SSA countries by Wiysonge et al. (2012) and other cross sectional studies conducted by Kidane et al. (2008), Elizabeth et al. (2003) and Ibnouf et al. (2007). These studies declared that maternal education was a significant predictor of completeness of immunization, in which highly educated mothers will be more aware of the importance of immunization [13, 18, 20, 22, 25, 27, 29].

In addition, educated women may choose health care services that generate better health. This may be because education may provide greater knowledge of the health care utilization and the ability to respond to new knowledge more rapidly [42–44].

In this study, wealth index was one of the most predictor variables of childhood full immunization status. Children belonging to wealthier families may be more likely to receive missing doses of vaccines when attending a health care facility than children from poor households. This finding is consistent with previous studies which had shown that there is statistically significant association between childhood vaccination completion rates and household wealth index; the higher the family wealth index the increased chance of being fully immunized children’s [13–15, 23, 28, 45].

It might be due to the fact that children who are from poor homes find it difficult to be reached by the health workers and also poor parents may encounter barriers to reach health facility compared to rich parents’ children. Case et al. (2002) found that parent’s long run income is important for the child’s health [46]. In addition, higher incomes are associated with better health seeking practices and health status [47].

The number of under 5 years children in the households was also a significant predictor of childhood immunization. As the number of under 5 years children in the household increases the chance of the last child to be fully vaccinated decreases. This finding corroborates with is Elizabeth et al’s (2003) study where children born to parents having one under five children, those children born to families with 2 or 3 children have lesser chance of being fully immunized [25]. Also Alister et al. (2007) revealed that Children born from larger families showed a low vaccination uptake [23].

A possible explanation for the incomplete immunization of the last child among parents having many under five children is that, they may develop confidence and may believe that modern health care is not as necessary due to the experience and knowledge accumulated from previous children’s. More over as the number of children in a household increases the available resource in the family may be depleted, parents may become busy in full filling the needs of their children. Another explanation could be the focus of mothers will tend to decrease as they give birth for many children.

Exposure to media was also a significant predictor of childhood immunization. As compared to children whose families reported lack of exposure to media (utilization of TV/Radio at least once a week), those children whose families having exposure to media had higher probability of completing their vaccination. This finding is consistent with the study conducted in 24 SSA countries where maternal access to media reduced the odds of a child being unimmunized [13]. This may be explained by the effectiveness of media in information dissemination. In addition, media could facilitate behavioral changes allowing for the adoption of different behaviors.

As expected, geographic region of the study participants was found to be statistically significant explanatory variable. Children from Addis Ababa City, Tigray, Amahara, Somali, BEGU regions and Dire Dawa City were more likely to be fully immunized than children from Affar region. This finding is consistent with the findings of previous studies conducted by Lerebo (2010), which disclosed that; in comparison to children whose region of residence was Affar region, children from Addis Ababa and Dire Dawa City, Tigray, Harari, Amahara, Gambella, SNNP, Oromia, Somalia and BEGU regions had higher chance of being fully immunized [26]. Similarly, different studies conducted elsewhere had found the significant effect of regional variations on childhood full immunization status [14, 23, 31–33]. This may be due to the fact that the 9 regional states and 2 city administrative councils found in Ethiopia consists of different religious, cultural, population size, geographic nature and levels of development. This could be linked with differences in vaccine supply, availability of health care providers and accessibility of health facilities. Hence, these regional divergences tend to affect the range of child immunization across the country.

Although the finding was consistent with previous studies conducted on determinants of childhood immunization and found geographic region as a strong significant predictors of childhood full immunization status with a larger number of AOR, in this study the magnitude is significantly reduced. This may be due to the nature of the analysis technique used in this study which considers the group effect and controls the inflation of the difference in magnitude.

Finally the study finding had also revealed that community maternal ANC services utilization was a significant predictor of childhood full immunization status. Accordingly, children of mothers residing in communities possessing higher proportion of maternal ANC services utilization had higher odds of being fully immunized than their counter parts residing in low maternal ANC services utilization communities.

Even though, there were no predictor variables used in the analysis to directly measure the availability of health facilities in the communities, maternal community ANC services utilization could be a proxy indicator for the availability of health facilities in these communities. Hence, the high proportion of community ANC rates may indicate the availability of health facilities in the community. This finding is similar with the findings of Antai (2010) where children of mothers residing in communities possessing higher proportion of maternal ANC services utilization had higher odds of being fully immunized [32].

This could be justified as; women lacking prenatal care are less likely to be informed of the importance of childhood immunization and other health promoting programs. Another possible explanation for this finding may be the increased confidence in the value of child immunization and institutional delivery amongst mothers who attended ANC services and amongst those who delivered in health facilities which may be developed from counseling during the ANC visits. This is further related with the fact that living in communities having higher proportion of maternal health seeking behaviors would be an influential factor. Women residing in the same community tend to behave or practice in the same way than women from different areas as the influence of sharing same sources of information, resources, culture and others.

Unlike what has been documented elsewhere [6, 20, 23, 29, 30], this study did not found significant association between childhood full immunization and maternal place of residence. This may be due to the fact that the small sample size they used [8, 20, 29, 33] and the time in the study was conducted by itself may be a factor [23]. Nevertheless, the study finding is consistent with the study conducted in central Ethiopia by Etana et al. (2012) and the study conducted in Nigeria by Aremu et al. (2010), in which both studies found non-significant association between likelihood of childhood full immunization and place of residence [17, 33] and this needs further investigation. The results of the random parts had shown a strongly significant variance remaining between communities even after simultaneous adjustment of individual and contextual level factors indicating contextual level factors were likely to influence immunization uptake. This finding was consistent with other multilevel studies conducted to examine determinants of childhood full immunization in Nigeria and 24 SSA countries [14, 19, 31–33]. This might be justified by the existence of difference in social norms, cultural beliefs, geographic, health service quality and coverage and other infrastructures.

The findings from this study were not without limitations and should be noted. First, the analysis was conducted using potential predictor variables extracted from the EDHS 2011. But variables other than mentioned in the DHS data set would be also likely to be important determinants of full immunization among children age 12–59 months. Some of these may includes distance to immunization centers and quality of immunization services. Secondly, given that the information on childhood full immunization was recorded retrospectively (using immunization card/maternal verbal response) it is highly prone to recall bias. Thirdly, the analyses were conducted using a data collected by a cross sectional survey, which further creates a problem of making causal inferences. Hence there is a need to verify the validity of the observed relationships using longitudinally collected data at different points of time.

Despite the limitations mentioned above, the study has numerous strengths. First, the data used in this analysis was the most recent, nationally representative and large sample of population based survey which covers across all regions and city administrative of the country. Second, the DHS surveys are similar in design having standard variables that are comparable across settings. Hence the finding could be generalized to other developing countries. Third, above all, unlike previous studies conducted using the DHS data in identifying determinants of childhood immunization, this study is unique in a way that advanced statistical technique called the multilevel modeling analysis was used which takes into account the nested nature of the DHS data, thus allowing for the clustering effect of the outcome variable to be examined which is an important phenomena that has to be considered.

## Conclusions

Even though there was an incremental trend over the past two DHS reports in childhood full immunization coverage in Ethiopia the coverage was still low (i.e. 26 %). This population based multilevel modeling analysis of childhood full immunization adds to our understanding of health service, socioeconomic, demographic and geographic variations associated with coverage of childhood immunization in Ethiopia. Individual level characteristics which includes; child place of delivery, maternal education, household wealth index, the number of under 5 years children in the household and household media exposure were found to be strong predictors of childhood immunization. Similarly, community level variables like; geographic region and community maternal ANC utilization were found to have significant association with childhood full immunization. The analysis result had also revealed that community level random effects remained significant after controlling for both individual and contextual level variables, indicating that immunization uptake significantly depends on community contexts. Hence, utilizing multilevel in determining the effect of both individual and contextual level factors simultaneously had brought an important output which may help planners, policy and decision makers to emphasize on individuals and communities in which they live.

## Abbreviations

AIC: Akakie Information Criterion
ANC: Antenatal Care
AOR: Adjusted Odds Ratio
BCG: Bacille Calmette Guérin
BEGU: Beneshangul Gumuz
CI: Confidence Interval
COR: Crude Odds Ratio
CSA: Central Statistics Authority
DHS: Demographic and Health Survey
DPT: Diphtheria-Pertussis-Tetanus
EDHS: Ethiopian Demographic and Health Survey
EPI: Expanded Programme on Immunization
FMoH: Federal Ministry of Health
Hep B: Hepatitis B
HSDP: Health Sector Development Programme
ICC: Intra Class Correlation
IQR: Inter Quartile Range
MDGs: Millennium Development Goals
MEASURE: Monitoring and Evaluation to Assess and Use Results
MLRA: Multilevel Logistic Regression Analysis
NGOs: Non-Governmental Organizations
OPV: Oral Polio Vaccine
PCV: Pneumococal Conjugate Vaccine
PCV: Proportional Change in variance
SE: Standard Error
SNNP: Southern Nations Nationalities and Peoples
TT: Tetanus Toxoid
VIF: Variance Inflation Factor
VPDs: Vaccine Preventable Diseases
WHO: World Health Organization

## References

[1] World Health Organization (WHO). Immunization service delivery: Expanded Programme on Immunization (EPI); Feb 2013 [Access Date Feb 14, 2013] Available from: http://www.who.int/immunization_delivery/en/.

[2] Centers for Disease Control and Prevention (CDC). Atlanta. Immunization schedules: immunization schedules for infants and children in easy-to-read formats; Feb 2013 [Access Date Feb 14, 2013] Available from: http://www.cdc.gov/vaccines/schedules/easy-to-read/child.html#print.

[3] WHO, UNICEF, World Bank. State of the world’s vaccines and immunization, 3rd ed. Geneva, World Health Organization, 2009. [Access Date Sept 27, 2015]. Available from http://apps.who.int/iris/bitstream/10665/44169/1/9789241563864_eng.pdf.

[4] WHO (2010). Vaccine preventable diseases monitoring system global summary: immunization, vaccines and biologicals.

[5] Federal Ministry of Health Ethiopia (2006). Ethiopia expanded program on immunization 2006–2010 multiyear plan. Addis Ababa.

[6] UNICEF. Millenium Development Goals (MDGs). Reduce child mortality, [Access Date Feb 13, 2013], Available from : http://www.unicef.org/mdg/childmortality.html.

[7] Kidane T, Tekie M (2003). Factors influencing child immunization coverage in rural district of Ethiopia, 2000. Ethiop J Health Dev.

[8] World Health Organization Country Office (2011). Annual report, Ethiopia.

[9] CSA [Ethiopia], ICF International (2012). Ethiopia demographic and health survey 2011.

[10] Ndiritu M, Cowgill KD, Ismail A, Chiphatsi S, Kamau T, Fegan G (2006). Immunization coverage and risk factors for failure to immunize with expanded programme on immunization in Kenya after introduction of new haemophilus influenza type 6 virus and hepatitis 6 virus antigens. BMC Public Health.

[11] Edmunds WJ, Brisson M, Melegaro A, Gay NJ (2002). The potential cost effectiveness of a cellular pertussis booster vaccination in England and Wales. Vaccine.

[12] World Health Organization. Global Tuberculosis Control: surveillance, planning, financing. WHO Report 2007 WHO/HTM/TB/2007.376. No date [Access date Sept 27 2015]. Available from: http://apps.who.int/bookorders/anglais/detart1.jsp?sesslan=1&codlan=1&codcol=15&codcch=3659.

[13] Department of Health and Social Services. Gert Sibande District 1st Quarter Review 2007/2008. District Health Office. Ermelo: Department of Health and Social Services, Mpumalanga.

[14] Babalola S (2008). Determinants of the uptake of the full dose of DPT in northern Nigeria: a multi level analysis Maternal Child Health Journal.

[15] Babirye JN, Engebretsen IMS, Makumbi F, Fadnes LT, Wamani H, Tylleskar T (2012). Timeliness of childhood vaccinations in Kampala Uganda: a community-based cross-sectional study. PLoS One.

[16] Odiit A, Amuge B (2003). Comparison of vaccination status of children born in health units and those born at home. East Afr Med J.

[17] Etana B, Deressa W (2012). Factors associated with complete immunization coverage in children aged 12–23 months in Ambo Woreda, Central Ethiopia. BMC Public Health.

[18] Haque SMR, Bari W (2013). Positive role of maternal education on measles vaccination coverage in Bangladesh. Int J Psychol Behav Sci.

[19] Wiysonge CS, Uthman OA, Ndumbe PM, Hussey GD (2012). Individual and contextual factors associated with low childhood immunization coverage in Sub-Saharan africa: a multilevel analysis. PLoS One.

[20] Ibnouf A, Vanden B, Maarse J (2007). Factors influencing immunization coverage among children under five years of age in Khartoum State, Sudan: University of Maastricht. S Afr Fam Pract.

[21] Antai D (2009). Inequitable childhood immunization uptake in Nigeria: a multilevel analysis of individual and contextual. BMC Infect Dis.

[22] Munthali AC (2007). Determinants of vaccination coverage in Malawi: evidence from the demographic and health surveys. Malawi Med J.

[23] Roy, SG. “Risk F actors for Childhood Immunization Incompletion in Ethiopia” (2010). Public Health Theses. Georgia State University, Paper 90. [Accessed 2013 February 5]. Available from: http://digitalarchive.gsu.edu/iph_theses/90.

[24] International Institute for Population Sciences and Macro International (IIPS) (2008). National family health survey, 2005–2006.

[25] Luman ET, McCauley MM, Shefer A, Chu SY (2003). Maternal characteristics associated with vaccination of young children. Paediatrics.

[26] Lerebo W (2010). Religion, education and child immunization in Ethiopia: the impact of maternal religion and education level on the uptake of child immunization in Ethiopian community.

[27] Tagbo BN, Onwuasigwe C (2005). Missed immunization opportunities among children in Enugu. Niger J Paediatr.

[28] Sanou A, Simboro S, Kouyaté B, Dugas M, Graham J, Bibeau G (2009). Assessment of factors associated with complete immunization coverage in children aged 12–23 months: a cross-sectional study in Nouna district, Burkina Faso. BMC Int Health Hum Right.

[29] Kidane T, Yigzaw A, Sahilemariam Y, Bulto T, Mengistu H, Malay T (2008). National EPI coverage survey report in Ethiopia, 2006. Ethiop J Health Dev.

[30] Mathew JL (2012). Inequality in childhood immunization in India: a systematic review. Indian Pediatr.

[31] World Health Organization (2002). State of the world’s vaccines and immunization 2002.

[32] Antai D (2011). Rural urban inequalities in childhood immunization in Nigeria: the role of community contexts. Afr J Prm Health Care Fam Med.

[33] Aremu O, Lawoko S, Dalal K (2010). Childhood vitamin A capsule supplementation coverage in Nigeria: a multilevel analysis of geographic and socioeconomic inequities. The Scientific World Journal: TSW Child Health & Human Development.

[34] Snijders T, Bosker R (2003). Multilevel analysis an introduction to basic and advanced multilevel modeling.

[35] Goldstein H (2011). Multilevel statistical models. University of Bristol, UK.

[36] Merlo J, Yang M, Chaix B, Lynch J, Rastam L (2005). A brief conceptual tutorial on multilevel analysisin social epidemiology: investigating contextual phenomena indifferent groups of people. J Epidemiol Community Health.

[37] Darmawan I, Keeves JP (2006). Suppressor variables and multilevel mixture modeling. Int Educ J.

[38] Schwarz G (1978). Estimating the dimension of a model. Ann Stat.

[39] Akaike H (1974). A New look at the statistical model identification. IEEE Trans Autom Control.

[40] Angela O, Babatunde F, Akinwumi F, Edet E (2012). Immunization coverage in selected communities in the Niger Delta, Nigeria. World J Vaccine.

[41] Kumar D, Aggarwal A, Gomber S (2010). Immunization status of children admitted to a tertiary-care hospital of north India: reasons for partial immunization or non-immunization. J Health Popul Nutr.

[42] Grossman M (2005). ‘Education and nonmarket outcomes’, Working Paper 11582, National Bureau of Economic Research (NBER).

[43] Llena-Nozal A, van der Klaauw B, Lindebooma M (2009). Parental education and child health: evidence from a schooling reform. J Health Econ.

[44] Bloom SS, Wypij D, Das GM (2001). Dimensions of women’s autonomy and the influence on maternal health care utilisation in a north Indian city. Demography.

[45] Ndirangu J, Bärnighausen T, Tanser F, Tint K, Newell M-L (2009). Levels of childhood vaccination coverage and the impact of maternal HIV status on child vaccination status in rural KwaZulu-Natal, South Africa. Trop Med Int Health.

[46] Case A, Lubotsky M, Paxson C (2002). Economic status and health in childhood: the origins of the gradient. Am Econ Rev.

[47] Case A, Lee D, Paxson C (2008). The income gradient in children’s health: a comment on Currie, Shields and Wheatley price. J Health Econ.

